# Harnessing the metabolic modulatory and antioxidant power of 1-(3-Phenyl-Propyl) cyclopropane and melatonin in maintaining mango fruit quality and prolongation storage life

**DOI:** 10.1186/s12870-023-04485-4

**Published:** 2023-10-05

**Authors:** Emad Hamdy Khedr, Nagwa Khedr, Mohamed Abdel-Haleem

**Affiliations:** 1 Department of Pomology, Faculty of Agriculture, Cairo University, Giza, 12613 Egypt; 2 Department of Botany and Microbiology, Faculty of Science, Zagazig University, Zagazig, 44519 Egypt

**Keywords:** Chilling injury, Electrolyte leakage, Energy metabolism, Keitt, *Mangifera*, Shelf life

## Abstract

**Background:**

The aim of this study was to compare and investigate the effects of 1-(3-phenyl-propyl) cyclopropene (PPCP) and melatonin (MT) as anti-ethylene agents on postharvest senescence, quality, chilling tolerance, and antioxidant metabolism in the mango fruit cv. “Keitt”. The study involved exposing the fruit to 20 μL L^− 1^ PPCP or 200 μM MT, in addition to a control group of untreated fruit, before storing them at 5 ± 1 °C for 28 d. The findings revealed that the treatments with PPCP and MT were effective in reducing chilling injury and preserving fruit quality when compared to the control group.

**Results:**

The use of 20 μL L^− 1^ PPCP was an effective treatment in terms of mitigating chilling injury and preserving fruit quality for 28 d. This was attributed to the decrease in metabolic activity, specifically the respiration rate and the production of ethylene, which led to the maintenance of fruit firmness and bioactive compounds, energy metabolism, and antioxidant activity, such as ascorbic acid, total flavonoids, trolox equivalent antioxidant capacity, dehydroascorbate reductase, glutathione reductase activity, ATP, and ATPase activity. The study also found that the MT treatment at 200 μM was effective in reducing chilling injury and weight loss and improving membrane stability. Additionally, it led to a decrease in malondialdehyde content and electrolyte leakage, and the maintenance of fruit quality in terms of firmness, peel and pulp colour values for mango peel and pulp total carotenoid content, as well as phenylalanine ammonia lyase and tyrosine ammonia lyase activity. These findings indicate that PPCP and MT have the potential to be efficient treatments in maintaining mango quality and minimizing post-harvest losses.

**Conclusion:**

The utilisation of treatments with 20 μL L^− 1^ of PPCP or 200 μM MT was found to effectively preserve the postharvest quality parameters, in terms of bioactive compounds, energy metabolism, and antioxidant activity, of mangoes cv. “Keitt” that were stored at 5 ± 1 °C for 28 d.

## Background

*Mangifera indica*, commonly known as mango, is a fruit widely appreciated for its nutritional composition, appealing fragrance, colour and flavour [1]. Despite its widespread popularity, mango is highly perishable, with a limited post-harvest shelf life under normal conditions, owing to its high respiration and ethylene production rates, and climacteric ripening behaviour [2]. Mango ‘Keitt’ cultivar, a commercial variety known for its moderate peel thickness, aromatic profile, and high ratio of total soluble solids (TSS) to acidity. Temperature control is a critical aspect of post-harvest management of fruits and vegetables, where low temperature storage is commonly employed to reduce respiration rates, suppress ethylene production, prevent nutrient degradation, maintain texture, and inhibit microbial and insect pest growth [3]. Mangoes, being of tropical and subtropical origins, are particularly susceptible to chilling injury (CI) when subjected to low temperatures, making the use of cold storage unsuitable for this fruit [4]. CI is a physiological disorder that arises from oxidative stress and membrane damage caused by excessive production of reactive oxygen species (ROS) when exposed to temperatures below 10–13 °C. Cold stress can cause a shift in the balance of unsaturated to saturated fatty acids in cell membranes, resulting in decreased firmness and vulnerability to lipid peroxidation by ROS, ultimately leading to irreversible membrane damage and the manifestation of CI symptoms in the fruit. In mangoes, these symptoms include epicarp discoloration, sunken lesions, irregular ripening, reduced flavour and aroma, and increased susceptibility to decay [5].

Several methods have been attempted to alleviate the impact of CI on mangoes, but their effectiveness is often limited by certain drawbacks. For example, treatments such as chitosan and spermidine [6] and nitric oxide [7] have been shown to reduce the production of ethylene and CO_2_, but they have been observed to negatively affect the development of the fruit’s colour. On the other hand, methods such as low-temperature conditioning [8] and the use of methyl jasmonate have been found to enhance the ripening process [9], but their impact on CI management remains modest. Similarly, the use of oxalic and salicylic acids in treatment has been shown to have limited efficacy in controlling CI [10]. Therefore, there is a need to explore alternative methods that can effectively reduce CI symptoms without compromising the fruit’s quality attributes. Cyclopropene compounds are known for their non-toxic and effective nature as ethylene antagonists. Previous studies have confirmed the ability of cyclopropene to delay fruit ripening and prolong the postharvest storage life of crops in sealed environments [11]. Several other cyclopropene compounds with substituent groups on the cyclopropene ring have been synthesized and evaluated for their efficacy as anti-ethylene agents [12]. However, some of these compounds, such as 1-ethylcyclopropene and 1-propylcyclopropene, have been shown to be less effective than 1-MCP [13]. To overcome this limitation, long-chain cyclopropenes, such as 2-alkyl-2-cyclopropene-1-carboxylic acid ethyl ester [12] and N,N-dialkyl-(1-cyclopropenylmethyl) amine compounds, have been proposed as more versatile options for use in open field applications [14]. A novel compound, 1-(3-phenyl-propyl) cyclopropene (PPCP), has recently emerged as a potential alternative to 1-MCP. However, the effectiveness of PPCP as an ethylene antagonist has not been thoroughly evaluated and compared to other cyclopropene compounds. PPCP (C_12_H_14_) has a higher molecular weight (158.24) than 1-MCP (C_4_H_6_) with a molecular weight of 54.09, but its ability to delay fruit ripening and extend the storage life of postharvest crops in both glasshouses and open fields remains to be determined [15], Therefore, further research is needed to compare PPCP’s efficacy and its ability to inhibit ethylene production in fruit.

**Fig. 1:**
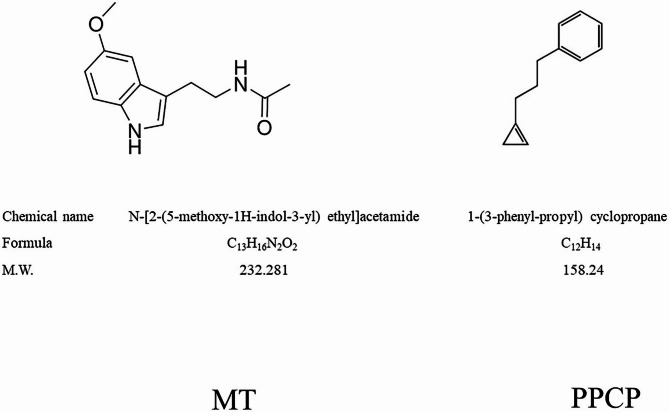
An illustration of the chemical structures and characteristics of melatonin (MT) and 1-(3-phenyl-propyl) cyclopropane (PPCP)

Melatonin (N-acetyl-5-methoxytryptamine) is effectively neutralizing reactive oxygen species (ROS) in a receptor-independent manner [16, 17]. Figure 1 illustrate the chemical structures and characteristics of MT and PPCP. In recent years, the use of melatonin for preserving the quality and extending the shelf life of fruits and vegetables has gained considerable interest. Melatonin has been found to delay senescence and enhance resistance to fungal decay, as reported in studies by Sagar et al. [18], Arnao et al. [17], and Hernandez-Ruiz et al. [19]. Furthermore, melatonin has been shown to effectively improve the chilling tolerance of various fruits such as pomegranate [20], peach [21, 22], litchi [23], plum [24], mango [25, 26], and banana fruit [27]. Recent studies have investigated the use of exogenous melatonin in maintaining postharvest physiological properties and prolonging the shelf life of fresh crops. Hu et al. [28] demonstrated that postharvest immersion treatment of melatonin at doses ranging from 0.05 to 0.5 mM was effective in reducing starch hydrolysis and ethylene production in banana fruit, resulting in delayed ripening.

To the best of our knowledge, no previous research has been conducted to compare and evaluate the efficacy of applying PPCP and MT to enhance the chilling tolerance of ‘Keitt’ mango fruit regarding bioactive compounds, energy metabolism, and antioxidant activity. The purpose of this study is to investigate the impact of exogenous treatments with PPCP and MT on the storage life and chilling tolerance of ‘Keitt’ mango. The study aims to examine the effects of these treatments on energy metabolism and tissue changes of mango fruit stored at 5 ± 1 °C for four weeks.

## Materials and methods

### Mango materials and chemicals

The mango used in the study were obtained from a local grower in Beheira Governorate (30°15’44.7"N 30°31’48.0"E), Egypt, and were of the mature-green stage (*Mangifera indica* L. cv. Keitt). The fruits were carefully selected based on size, firmness, and overall appearance, as outlined by Khedr [29]. The chosen mangoes had an average weight of 350–450 g and a firmness level of 69.89 ± 5.21 N. They were also free of any visible damage and had an even colouration on the peel. Upon being sourced, the mangoes were promptly transported to the post-harvest laboratory at the Faculty of Agriculture, Giza, Egypt, where the experiments were conducted. All the necessary chemicals for the preparation of MT and PPCP treatments were sourced from Sigma-Aldrich Inc. located in St. Louis, MO, USA and Erum Biotechnologies Inc. situated in Suwon, Korea.

### Experimental design and treatments

In accordance with our preliminary study employing response surface methodology (RSM), we determined the optimal concentrations of 20 μL L^− 1^ PPCP and 200 μM MT for further investigation. The selected mangoes were immediately transported to the post-harvest laboratory at the Faculty of Agriculture, Giza, Egypt, after being thoroughly cleaned by immersion in a 0.05% sodium hypochlorite solution for 3 min and rinsed with distilled water. The cleaned fruits were then divided into three groups, with each group consisting of 150 fruits. The first group was dipped in distilled water (as the control group). The PPCP treatment was conducted by preparing a solution of 20 μL L^− 1^ PPCP via reaction of its precursor with tetrabutylammonium fluoride in dimethyl sulfoxide. The mangoes were placed in 15 sealed containers (20 L), each contained within an acrylic chamber of 0.9 m^3^, and exposed to the PPCP solution in these containers. The preparation of PPCP involved the use of 528.59 mg of precursor. The chamber was tightly sealed, and the hexane solution containing the PPCP was agitated and allowed to completely vaporize by pumping air into it for 30 min. The treatment was conducted for 24 h at 20 °C, after which the chambers were opened, and the air was circulated. The MT treatment was executed by submerging the fruit in a 200 μM solution for 120 min, with low light exposure at a temperature of 20 °C. The concentration and duration were determined based on previous screening studies [2]. Following the MT solution or distilled water removal, the fruits were air-dried for 2 h at room temperature. All treatments were stored at 5 ± 1 °C and 85–90% relative humidity. The samples were taken every 7 days from cold storage, followed by 3 days at 20 °C. Then, all the parameters were evaluated.

### Evaluation of fruit quality parameters

#### Chilling injury (CI) index

The method for determining the CI index, which was modified slightly from the approach described by Cantre et al. [4], involved evaluating 90 fruits, with 30 fruits per experimental treatment. The symptoms under examination were darkening of lenticels, greyish scald, and pitting. To assess the severity of these symptoms, a visual evaluation was conducted using a CI index and a 5-point scale based on the percentage of affected tissue. The scale ranged from 0 (no tissue injury) to 4 (76–100% tissue injury). The CI index was calculated as a percentage, determined through three sets of 10 fruit samples, each obtained from independent replicates. The calculation was performed using the formula:

$${\rm{CI}}\,{\rm{index}} = \frac{{\sum {({\rm{rank}}\,{\rm{score}}\, \times \,{\rm{number}}\,{\rm{of}}\,{\rm{fruit}}\,{\rm{in}}\,{\rm{each}}\,{\rm{CI}}\,{\rm{rank}})} }}{{{\rm{total}}\,{\rm{number}}\,{\rm{of}}\,{\rm{fruit}}\,{\rm{observed}}}}$$

#### Ethylene production and respiration rate

The respiration rate and ethylene synthesis were evaluated by placing mangoes in sealed 2-liter glass containers under controlled conditions for a 24-hour period. The CO_2_ level was analyzed using gas chromatography (Servomex 1400, Washington, USA). The respiration rate of the fruit was calculated as the rate of CO_2_ release per unit weight per hour, expressed in μg CO_2_ kg^–1^ s^–1^. Ethylene production was quantified by using the same container and methodology as for the respiration rate measurement, with an ethylene analyzer (Model F-950, Felix Instruments, Camas, WA, USA). The results of the ethylene synthesis were expressed in terms of ng C_2_H_4_ kg^–1^ s^–1^, as reported by Khedr [30]. The calculations for both the respiration rate and ethylene synthesis were performed using the following formulas:

$${\rm{Respiratation}}\,{\rm{rate}} = \frac{{{\rm{carbon}}\,{\rm{dioxide}}\,{\rm{produced}}\, \times \,{\rm{head}}\,{\rm{space}}\,{\rm{volume}}}}{{{\rm{fruit}}\,{\rm{weight}}\, \times \,{\rm{incubation}}\,{\rm{period}}}}$$

$${\rm{Ethylene}}\,{\rm{production}} = \frac{{{\rm{ethylene}}\,{\rm{produced}}\, \times \,{\rm{head}}\,{\rm{space}}\,{\rm{volume}}}}{{{\rm{fruit}}\,{\rm{weight}}\, \times \,{\rm{incubation}}\,{\rm{period}}}}$$

#### Weight loss

The weight of the fruits were determined at the beginning of storage and at each subsequent measurement day using a digital scale. This information was used to calculate the weight loss percentage by comparing the final weight to the initial weight, as described in the method reported by Khedr [31].

#### Fruit firmness

The firmness of the fruit was measured using an 8-mm diameter probe attached in a fruit pressure tester (Mecmesin, force-torque test, England), using the method provided by Khedr and Al-Khayri [2]. The measurement was taken at two opposing points along the equatorial region, penetrating to a depth of 5 mm, using a sample of three fruit. This process was repeated three times to obtain three separate replicates. The results were recorded in units of Newton (N).

#### Peel and pulp colour

The colour of the fruit peel was assessed based on the L^*^, a^*^, and b^*^ colour concept, where L^*^ reflects the degree of lightness or darkness, a^*^ indicates the level of greenness (-) or redness (+), and b^*^ shows the extent of yellowness (+) or blueness (-). These measurements were performed using a Minolta CR-400 Chroma Meter (Konica Minolta Sensing Inc., Osaka, Japan) and the CIE system. The equatorial axis of two opposing sides of each fruit was analyzed and the observations were recorded in triplicate using a sample of 3 fruit per replicate, as described in the method reported by Bhardwaj et al. [32].

The total carotenoid content of the fruit pulp was calculated by extracting pigments from 0.5 g of fruit pulp samples using N,N-dimethylformamide as the solvent. The extracts were filtered, and the absorbance was measured at 470, 647, and 663 nm using a spectrophotometer (6300 UV/Visible Spectrophotometer, Jenway, Cole-Parmer Ltd., United Kingdom). The total carotenoid content was determined according to the method reported by Liu et al. [1], using the following formula: Total Carotenoids (μg g ^− 1^) = 1000 A_470_ – 3.27 (12.21 A_663_ − 281 A_647_) – 104 (20.13 A_647_ − 5.03 A_663_).

#### Malondialdehyde (MDA) content

MDA was measured using a modified version of the thiobarbituric acid (TBA) technique described by Xu et [33]. A sample of 3 g of mango tissue was mixed with 30 mL of 80:20 (v/v) ethanol and water solution and then centrifuged at 3000 g and 4 °C for 10 min. From the supernatant, 1 mL was combined with either 1 mL of solution A, contained 20% trichloroacetic acid (TCA) and 0.01% butylated hydroxytoluene (BHT), or solution B, composed of 20% TCA, 0.01% BHT and 0.65% TBA. After being shaken and heated (95 °C for 25 min), the solutions were then centrifuged. The absorbances were read at 440, 532, and 600 nm using a spectrophotometer (6300 UV/Visible Spectrophotometer, Jenway, Cole-Parmer Ltd., United Kingdom). The MDA equivalents were calculated based on the formulas: (1) A = [(Abs 532_+ TBA_) – (Abs 600_+ TBA_) – (Abs 532_–TBA_ – Abs 600_–TBA_)], (2) B = [(Abs 440_+ TBA_ – Abs 600_+ TBA_) x 0.0571], (3) MDA equivalents (nmol mL^–1^) = (A – B/157,000) x 10^6^.

#### Electrolyte leakage

The assessment of electrolyte leakage (EL) was performed using a modified version of the method described by Khaliq et al. [34]. Ten circular pieces (0.5 cm^2^) were cut from the peel and flesh of the fruit using a stainless-steel instrument. The samples were then rinsed three times and soaked in 25 mL of distilled water at 25 °C for 30 min. The initial electrolyte levels in the solution were measured using a conductivity meter (HI 98311; Hanna Instruments, Woonsocket, RI). The solution was then subjected to a temperature of 121 °C for 10 min to extract all the electrolytes, after which it was cooled, and the electrolyte level was re-measured. The percentage of EL was calculated as follows: EL% = (initial electrolyte levels / total electrolyte levels) x 100.

#### Analysis of bioactive compounds

The quantification of ascorbic acid (AsA) in mango samples was performed using a titration method that employed 2,6-dichlorophenol, as reported by Cao et al. [21]. To begin, mango samples were blended with a 2% oxalic acid solution to produce a final volume of 100 mL. This homogenate was filtered, and 10 mL of the filtrate was titrated with a standardized solution of 2.6-dichloroindophenol. The end point of the titration process was recognized by the change in colour to pink. The AsA content was then calculated based on the following formula:

$$\text{A}\text{s}\text{A} \text{c}\text{o}\text{n}\text{t}\text{e}\text{n}\text{t}=\frac{(\text{N}-{\text{N}}_{1})}{\text{T}} \times \frac{\text{C} \times \text{D} }{\text{W}} \times 100$$

The concentration of AsA in the fruit samples is represented in terms of mg per 100 g of sample. The volume of the titrating solution, indicated as N, is recorded in mL. The volume of titrating solution used in the blank titration, designated as N1, is expressed in mg. The relationship between the volume of the titrating solution and the amount of ascorbic acid present, denoted as C, is established as 1 mL of titrating solution per milligram of ascorbic acid. The volume of sample solution used during the titration process, indicated as T, is measured in mL. The final volume of the diluted sample solution, represented as D, is also recorded in mL. The weight of the sample is recorded in g, indicated as W.

A procedure based on the method described by Moo-Huchin et al. [35] was employed to quantify the total flavonoids content (TFC) in mango fruit. The first step was to prepare a methanol extract (ME) of the mango pulp. This was done by homogenizing 1 gram of mango pulp in 5 mL of methanol, then subjecting the homogenate to sonication for 30 min at 30 °C. The solution was then stored at 4 °C for 24 h and the process repeated for 3 days with the solvent being changed every 24 h. The final concentrated methanol extract was then stored in darkness at -20 °C. The extract was then diluted at a ratio of 1:3 with methanol, followed by mixing with 2 mL of deionized water and 150 mL of a 5% solution of NaNO_2_ for 5 min. Then, 150 mL of a 10% AlCl_3_ solution in methanol was added to the mixture, allowed to stand for 1 min at 25 °C, followed by the addition of 1 mL of 1 M NaOH. The volume was then adjusted to 5 mL with deionized water, shaken, and the absorbance was read at 415 nm to determine the TFC.

#### Trolox equivalent antioxidant capacity (TEAC)

TEAC assay was performed with a few modifications using the 2,2-azino-bis (3-ethylbenzothialozine-6-sulphonic acid), ABTS radical (ABTS^*+^), as reported by Re et al. [36]. The extract was added to a 7 mM ABTS^*+^ radical solution produced with an oxidizing agent (2.45 mM potassium persulphate). The reaction mixture was then incubated for 10 min at 30 °C, and the absorbance at 734 nm was measured after that time. The collected data was used to compute the scavenging activity using the following formula:

$$\text{T}\text{E}\text{A}\text{C} =\frac{({\text{A}\text{B}\text{S}}_{\text{C}\text{o}\text{n}\text{t}\text{r}\text{o}\text{l}}-{\text{A}\text{B}\text{S}}_{\text{S}\text{a}\text{m}\text{p}\text{l}\text{e}})}{{\text{A}\text{B}\text{S}}_{\text{C}\text{o}\text{n}\text{t}\text{r}\text{o}\text{l}}} \times 100$$

#### Phenylalanine ammonia lyase (PAL) and tyrosine ammonia lyase (TAL) activity

TAL (EC 4.3.1.25) and PAL (EC 4.3.1.5) enzymatic activity were measured using a modified procedure based on Khan et al. [37]. Mango fruit tissue (1 g) was homogenized with an ice-cold Tris-HCl buffer (50 mM, pH 8.5) containing 5% (w/v) polyvinylpolypyrrolidone (PVPP) and 14.4 mM β-mercaptoethanol to obtain a preliminary extract. The extract was then centrifuged at 10,000 g for 20 min at 4 °C, and the supernatant was used to measure the activity of PAL and TAL.

For the measurement of PAL activity, a reaction mixture was prepared by combining 800 μL of Tris-HCl buffer (0.5 mM, pH 8.0), 600 mM L-phenylalanine, and 100 μL of enzyme extract. The mixture was then incubated at 40 °C for 1 h. After stopping the reaction with 100 μL of 5 N HCl, the absorbance of the resultant solution was measured at 290 nm. Enzyme activity was calculated from the obtained data and expressed as U kg^-1^ of protein. One unit (U) of enzyme activity was defined as the amount of enzyme required to produce one nanomole of cinnamic acid per minute.

To measure the activity of Tyrosine Ammonia Lyase (TAL), a reaction mixture of 800 μL of Tris-HCl buffer (0.5 mM, pH 8.0), 100 μL of 5.5 μM L-tyrosine, and 100 μL of enzyme extract was produced. The mixture was incubated at 40 °C for 1 h. To halt the process, 100 μL of 5 N HCl was added, and the solution’s absorbance was measured at 333 nm. The information gathered was utilized to compute enzyme activity, which was represented as U kg^-1^ of protein. A unit of enzyme activity was defined as the amount of enzyme required to create one n mole of coumaric acid per minute.

#### Dehydroascorbate reductase (DHAR) and glutathione reductase (GR) activity

To determine DHAR and GR, the extract was obtained by homogenizing 1 g of fruit tissue in 0.1 M potassium phosphate buffer that has been pre-cooled (pH 7.0). After centrifuging the mixture at 12,000 g for 20 min at 4 °C, the supernatant was collected and used to measure antioxidant enzyme activity (GR and DHAR). DHAR (EC 1.8.5.1) activity was determined using a modified version of the dehydroascorbate reduction method published by Bhardwaj et al. [25]. The reaction mixture contained 0.1 M phosphate buffer (pH 7.0), 1 mM EDTA, 15 mM reduced glutathione (GSH), 2 mM dehydroascorbate, and the enzyme extract. The reaction was monitored at 265 nm for one minute, and the change in absorbance was used to compute DHAR activity. The results were expressed in terms of U kg^-1^ of protein. A unit of DHAR activity was defined as the quantity of enzyme required to create 1.0 mol of reduced ascorbate per minute.

Glutathione reductase (GR, EC 1.6.4.2) activity was measured by measuring NADPH oxidation, as described by Bhardwaj et al. [25], with some changes. 50 mM phosphate buffer (pH 7.0), 3 mM EDTA disodium salt, 0.1 mM NADPH, 1 mM oxidized glutathione (GSSG), and an enzyme extract were added to the reaction mixture. For one minute, the reaction was measured at 340 nm, and the change in absorbance was used to calculate GR activity. The findings were presented in units per Kg of protein. A unit of GR activity is defined as the amount of enzyme necessary to catalyze the oxidation of 1 nmol of NADPH per minute.

#### ATP and ATPase activity measurement

With slight modifications, the procedure described by Jin et al. [38] was utilized to determine ATP and H^+^-ATPase (EC 3.6.3.6) activity extract in triplicate, with each replication consisting of three fruits. 50 mM Tris-HCl buffer (pH 8.0), 0.25 M sucrose, 0.3 M mannitol, 0.5 g L^-1^ polyvinyl pyrrolidone, and 1 M EDTA were used to make a buffer. The fruit tissue was then homogenized in 10 ml of pre-cooled extraction buffer. The homogenate was centrifuged at 5,000 x g for 10 min at 4 °C, and the supernatant was collected. The leftover sediment was washed using a washing solution of 10 mM Tris-HCl, 0.25 M sucrose, 1 mM EDTA, and 0.3 M mannitol. The reaction was then terminated with the addition of 30 mM trichloroacetic acid (TCA), and the absorbance was recorded at 660 nm. One unit of activity was defined as the release of 1 μM phosphorus per minute.

#### Protein concentration

The protein content of the extract was assessed using Bhardwaj et al. [26] technique, with minor changes. 1 g of fruit tissue was homogenized in 10 mL of cold 50 mM sodium phosphate buffer with PVPP, 0.5 mM magnesium chloride, and 2 mM phenylmethylsulfonyl fluoride. The mixture was centrifuged for 15 min at 12,000 g at 4 °C, and the supernatant produced served as the crude extract. After 5 min of reaction, 5 mL of Bradford’s reagent was added to 0.1 mL of crude extract, and the absorbance was measured at 595 nm. Using a calibration curve of bovine serum albumin, the absorbance data was utilized to quantify the protein content.

### Statistical analyses

The study was carried out utilizing a completely randomized design (CRD) with three replicates. The data was reported as the average of three measurements with standard error (SE). MSTAT-C software was used to analyze the data using a one-way analysis of variance (ANOVA) with a probability threshold of 0.05. The Duncan multiple range post-hoc test was then used to further distinguish the means [39]. XLSTAT (Addinsoft, New York, NY, USA) was used to perform principal component analysis (PCA) to determine connections between variables and fruit quality measurements. Pearson’s correlation analysis was also utilized to explore the associations between mango quality parameters during storage at P values of 0.05 and 0.01.

## Results

### Chilling injury (CI) index

**Fig. 2:**
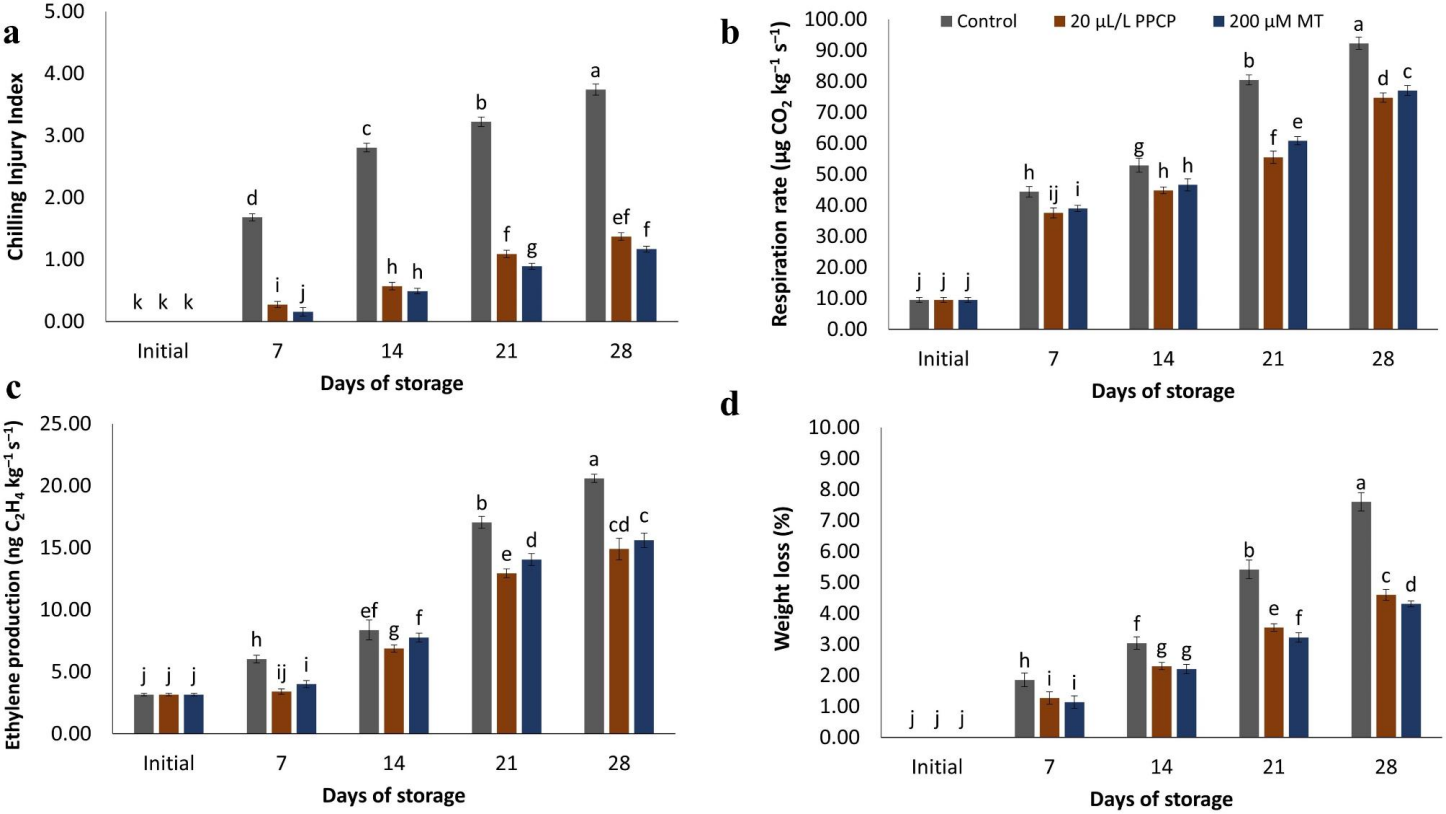
Effect of PPCP (1-(3-phenyl-propyl) cyclopropane) and melatonin (MT) postharvest treatments on the chilling injury index (**a**), respiration rate (**b**), ethylene production (**c**) and weight loss (**d**) of ‘Keitt’ mango fruit stored at 5 ± 1 °C for 28 d. Vertical bars indicate the standard error of the means with different letters indicating significant variance (*p* ≤ 0.05) between means, as determined by Duncan’s multiple range test

The indications of CI, which appeared as deep brown lesions and pitting, progressed in all groups, albeit to varying degrees of severity (Fig. 2a). After 7 d of cold storage (5 ± 1° C) plus 3 d shelf-life period at 20 °C, control had obvious signs of CI. While CI signs were more noticeable in all treatments at 14 d of storage, followed by 3 d shelf life under 20 °C. However, the MT and PPCP treatments significantly (*p* ≤ 0.05) suppressed the CI incidence. Treatment with 200 μM MT resulted in the lowest rate of CI (1.17) after the 28-day storage period at 5 ± 1 °C, whereas the CI reached 3.74 in untreated fruit on the same day.

### Ethylene production and respiration rate

After 21 days of cold storage, there was a climacteric increase in respiration rate (Fig. 2b) and ethylene production (Fig. 2c) in the control group. However, as the experiment progressed, the rate of ethylene generation and respiration rose progressively. Both the PPCP and MT treatments inhibited this rise when compared to the control. Furthermore, when compared to all other treatments, the 20 μL L^− 1^ PPCP treatment was the most successful in lowering both respiration rate and ethylene generation after 28 days of observation.

### Weight loss

Weight loss increased with storage period; this increment was more noticeable in all treatments at 14 d of storage. All treatments used significantly reduced weight loss (%) compared to the control, as it reached its highest value of 7.61 ± 0.26% in control fruits but was significantly (*p* ≤ 0.05) lower in fruits treated by 200 μM MT and 20 μL L^− 1^ PPCP, being 4.31 ± 0.27, and 4.62 ± 0.23%, respectively (Fig. 2d).

### Fruit firmness

**Fig. 3:**
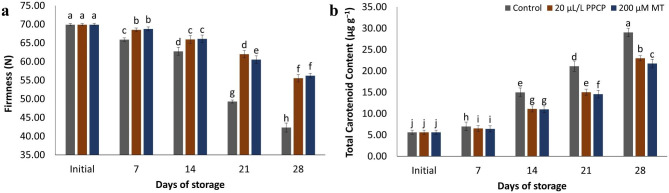
Impact of PPCP (1-(3-phenyl-propyl) cyclopropane) and melatonin (MT) postharvest treatments on the firmness (**a**), and total carotenoid content (**b**) of ‘Keitt’ mango fruit stored at 5 ± 1 °C for 28 d. Vertical bars indicate the standard error of the means with different letters indicating significant differences (P ≤ 0.05) between means, as determined by Duncan’s multiple range test

The firmness of the flesh is an essential measure of fruit quality. It is essential for all fruit chemical contents as well as fruit structure and handling operations. Fruit hardness steadily reduced as storage time increased (Fig. 3a). Fruit firmness was maintained across MT and PPCP treatments. Fruit treated with 200 μM MT and 20 μL L^− 1^ PPCP were substantially harder than control fruit, which softened at a faster pace. Fruit treated with 200 M MT had a firmness of 56.22 ± 0.97 N at the end of the storage period, whereas control fruit had a firmness of 42.33 ± 0.86 N.

### Peel and pulp colour

The results presented in Table 1 reveal that the colour of the peels was significantly affected by both the treatments and storage duration. There was a gradual increase in L^*^ values, followed by a decrease in the peels of all groups throughout the experimental period. However, no statistically significant difference (*p* ≤ 0.05) was observed at 14 days in all treated groups (Table 1). Subsequently, fruit treated with MT and PPCP exhibited higher, statistically significant L^*^ values compared to the untreated ones. At the end of the storage period, fruit treated with 200 μM MT displayed an L^*^ value of 51.58 ± 0.91, while control fruit exhibited an L^*^ value of 42.96 ± 0.76. The chromaticity a^*^ value gradually increased in both control and treated fruit, indicating a decrease in peel greenness because of prolonged storage (Table 1). The chromaticity b^*^ value gradually increased in both control and treated fruit, indicating an increase in yellowness in the peels as the fruit ripened (Table 1). The carotenoid content in the pulp, a pigment that contributes to the yellow colour, showed a continuous increase in parallel with the changes in chromaticity L^*^, a^*^, and b^*^ in the peels of both control and treated fruit (Fig. 3b). However, treatment with MT notably suppressed yellowing in the pulp.

**Table 1:** Effect of 1-(3-phenyl-propyl) cyclopropene (PPCP) and melatonin (MT) treatments on L^*^, a^*^, and b^*^ of mango cv. “Keitt” stored at 5 ± 1°C^z^

Treatments (T)	Days of storage at 5 °C					
	Initial	7	14	21	28	Mean (T)
***L*** ^*^ ***value***						
Control	56.32 ^c^	59.00 ^a^	57.18 ^b^	48.22 ^h^	42.96 ^i^	52.74 ^C^
20 μL/L PPCP	56.32 ^c^	57.25 ^b^	57.59 ^b^	52.60 ^e^	49.93 ^g^	54.74 ^B^
200 μM MT	56.32 ^c^	56.96 ^bc^	57.11 ^b^	54.33 ^d^	51.58 ^f^	55.26 ^A^
***a*** ^*^ **value**						
Control	-15.93 ^j^	-15.04 ^i^	-11.27 ^ef^	-6.43 ^b^	-3.52 ^a^	-10.44 ^A^
20 μL/L PPCP	-15.93 ^j^	-15.50 ^ij^	-12.53 ^g^	-10.84 ^de^	-9.86 ^c^	-12.93 ^B^
200 μM MT	-15.93 ^j^	-15.53 ^ij^	-13.58 ^h^	-11.56 ^f^	-10.55 ^d^	-13.43 ^C^
***b*** ^*^ **value**						
Control	30.66 ^d^	31.85 ^cd^	33.92 ^b^	15.36 ^e^	10.94 ^f^	24.55 ^C^
20 μL/L PPCP	30.66 ^d^	31.03 ^cd^	32.03 ^c^	33.52 ^b^	36.31 ^a^	32.71 ^A^
200 μM MT	30.66 ^d^	30.89 ^cd^	31.51 ^cd^	32.01 ^c^	34.67 ^b^	31.95 ^B^

^Z^Measurements were conducted every 7 d of 5 ± 1 °C storage followed by 3 d of shelf life at room temperature. Each value is the mean for three replicates. Values followed by different letters differ significantly (*p* ≤ 0.05). Small letters show difference between treatments during storage periods. Capital letters show difference between mean values for every treatment

### Malondialdehyde (MDA) content

**Fig. 4:**
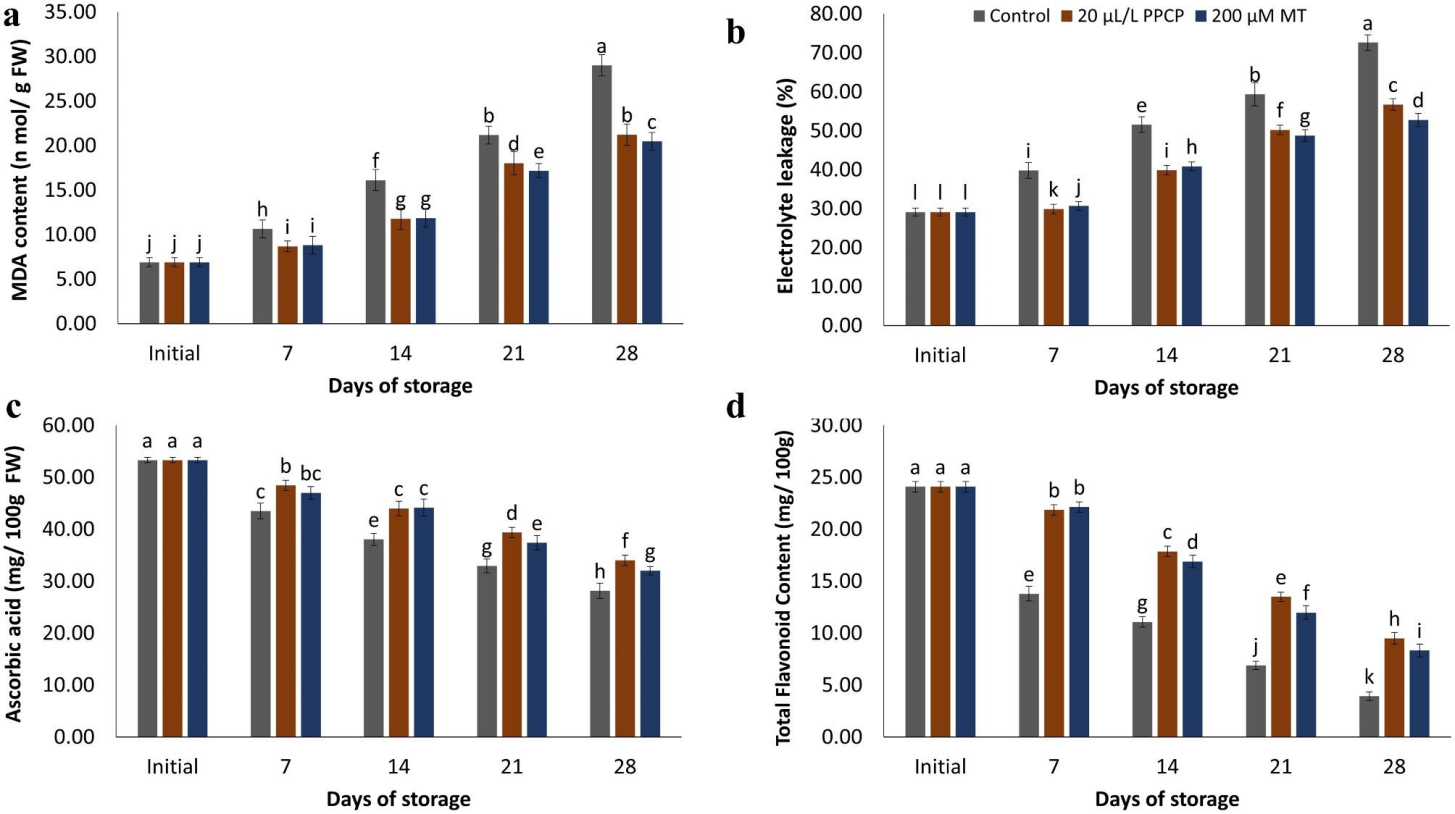
Influence of PPCP (1-(3-phenyl-propyl) cyclopropane) and melatonin (MT) postharvest treatments on the malondialdehyde (MDA) content (**a**), electrolyte leakage (**b**), ascorbic acid (**c**) and total flavonoid content (**d**) of ‘Keitt’ mango fruit stored at 5 ± 1 °C for 28 d. Vertical bars indicate the standard error of the means with different letters indicating significant variance (*p* ≤ 0.05) between means, as determined by Duncan’s multiple range test

The data shown in Fig. 4a show that the MDA concentration of mango fruit has been rising over time. When mango fruit was treated with 200 μM MT and 20 μL L^− 1^ PPCP, the MDA concentration increased less dramatically and remained lower than in the control. Furthermore, mango MDA concentration was lower in PPCP and MT-treated fruit than in the control group throughout the storage period (*p* ≤ 0.05). Throughout the storage period, MDA levels in all fruits increased steadily. Notably, even after 28 days of storage, treatment with 200 μM MT was able to maintain low MDA content (Fig. 4a).

### Electrolyte leakage (EL)

The EL values displayed a gradual increase during the 28 d of cold storage, particularly in the control fruit which exhibited significantly higher values than the other treatments (*p* ≤ 0.05), which may be related to a protective effect provided by the treatments (Fig. 4b). Among the treatments, 200 μM MT was found to be the most effective, showing the lowest EL values at the end of the cold storage period. The EL values of mangoes treated with 200 μM MT and 20 μL L^− 1^ PPCP were 52.80 ± 0.97%, and 56.75 ± 0.93%, respectively, while the control group showed 72.62 ± 1.13%.

### Analysis of bioactive compounds

As demonstrated in Fig. 4c, the AsA concentration was found to be significantly higher for all treatments than the control. The concentration of AsA steadily decreased with storage time. At the end of storage, the AsA content in the control treatments had declined by 53.77% as compared to the harvest day. The content of AsA in control treatments had dropped to 28.14 ± 0.68 mg/100 g FW after 28 d of storage at 5 ± 1 °C. All treatments used were found to significantly delay the decrease of vitamin C as compared to the control. Among the treatments, the highest amounts of vitamin C were retained in the mangoes treated with 20 μL L^−1^ PPCP throughout the entire storage period.

As illustrated in Fig. 4d, the estimated values of the total flavonoid content (TFC) revealed that all treatments applied maintained significantly higher values of TFC compared with the control fruit. The treatments of PPCP and MT were found to delay the decline of TPC during 28 days of cold storage. The highest content of total flavonoids content in fruit treated with 20 μL L^− 1^ PPCP was found to be 8.34 ± 0.57 mg/100 g after 4 weeks of storage at 5 °C. The lowest values were observed in the control fruit and were found to be 3.94 ± 0.47 mg/100 g after 4 weeks of storage at 5 °C.

### Trolox equivalent antioxidant capacity (TEAC)

**Fig. 5:**
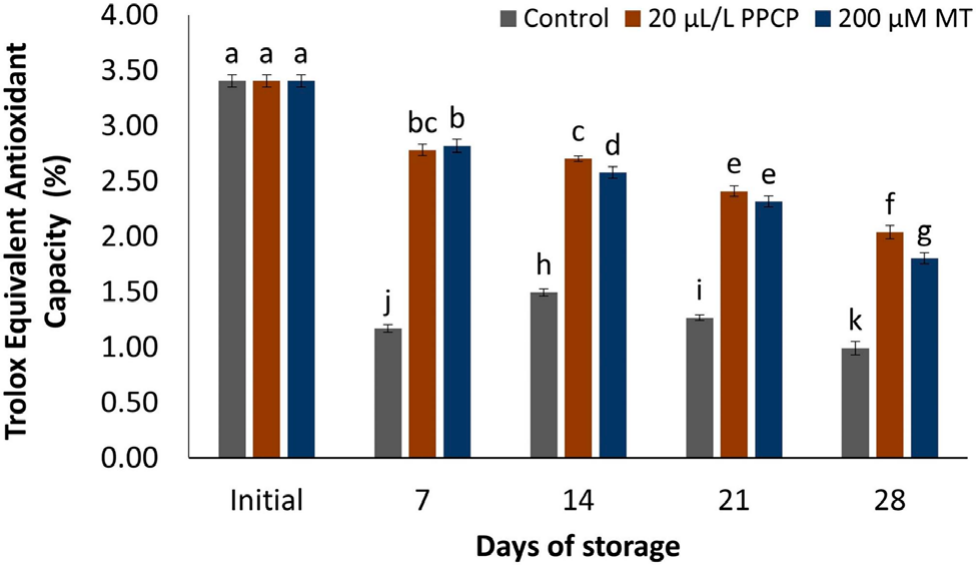
Influence of PPCP (1-(3-phenyl-propyl) cyclopropane) and melatonin (MT) postharvest treatments on the trolox equivalent antioxidant capacity (TEAC) of ‘Keitt’ mango fruit stored at 5 ± 1 °C for 28 d. Vertical bars indicate the standard error of the means with different letters indicating significant variance (*p* ≤ 0.05) between means, as determined by Duncan’s multiple range test

TEAC is used to evaluate a substance’s antioxidant capabilities. This value is determined by comparing the antioxidant activity of the substance being assessed to that of a standard antioxidant, Trolox. The TEAC values of mango fruit can vary depending on the variety of mango and the growing conditions. In general, as demonstrated in Fig. 5. the TEAC of untreated fruit decreased significantly after a 28-day storage period, whereas treated fruit exhibited improved TEAC scores. In most cases, there were no significant differences in TEAC between PPCP and MT-treated mango fruit.

### Phenylalanine ammonia lyase (PAL) and tyrosine ammonia lyase (TAL) activity

**Fig. 6:**
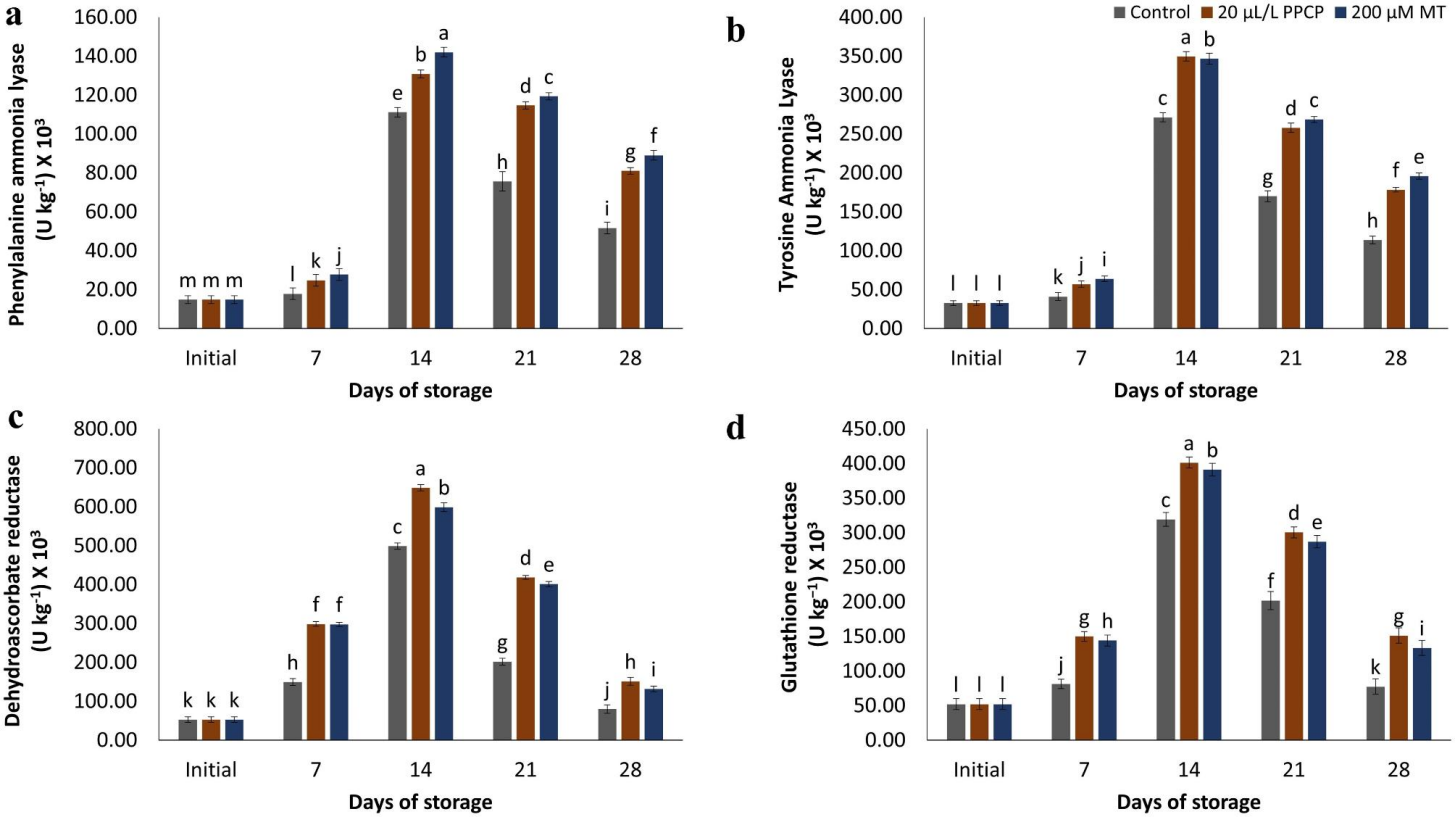
Effect of PPCP (1-(3-phenyl-propyl) cyclopropane) and melatonin (MT) postharvest treatments on the phenylalanine ammonia lyase (PAL) activity (**a**), and tyrosine ammonia lyase (TAL) activity (**b**), dehydroascorbate reductase (DHAR) activity (**c**) and glutathione reductase (GR) activity (**d**) of ‘Keitt’ mango fruit stored at 5 ± 1 °C for 28 d. Vertical bars indicate the standard error of the means with different letters indicating significant variance (P ≤ 0.05) between means, as determined by Duncan’s multiple range test

The activity of the enzymes PAL and TAL were observed to fluctuate throughout the storage period in the treated and control fruit. Initially, there was a gradual increase in the activity of both enzymes, however, towards the end of the storage period, a sudden decrease was observed in all treatments. Notably, the fruit treated with MT had much greater activity levels (*p* ≤ 0.05) of both PAL and TAL enzymes at 28 d of cold storage, as depicted in Fig. 6a and b. After a 4-week storage period at 5 ± 1 °C followed by 3 d at 20 °C, it was observed that the untreated fruit had the lowest activity of PAL and TAL enzymes, while the fruit treated with MT displayed the highest significant activity.

### Dehydroascorbate reductase (DHAR) and glutathione reductase (GR) activity

The activity of enzymes DHAR and GR displayed a gradual increase during the initial two weeks of storage, however, a sudden decrease was observed in all treatments under experimental conditions. The fruit treated with PPCP and MT maintained a notably higher activity of DHAR and GR enzymes in comparison to untreated mango fruit. Notably, PPCP treatment exhibited the highest significant values after four weeks of storage at a temperature of 5 °C, followed by three days at 20 °C, as illustrated in Fig. 6c,d.

### ATP and ATPase activity measurement

**Fig. 7:**
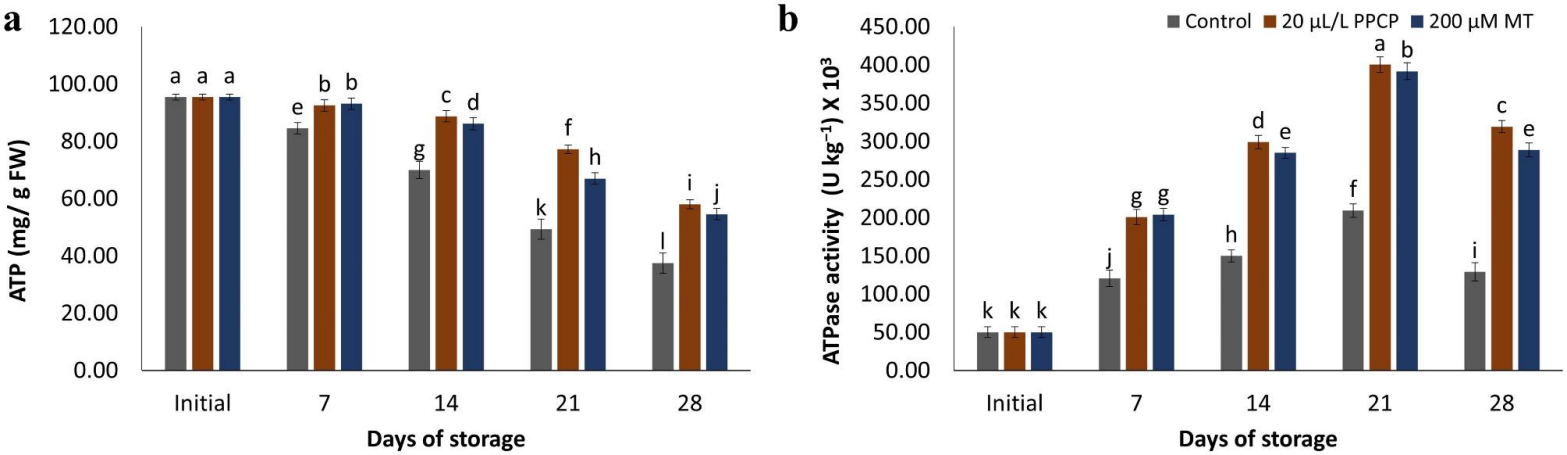
Impact of PPCP (1-(3-phenyl-propyl) cyclopropane) and melatonin (MT) postharvest treatments on the ATP (**a**), and ATPase activity (**b**) of ‘Keitt’ mango fruit stored at 5 ± 1 °C for 28 d. Vertical bars indicate the standard error of the means with different letters indicating significant variance (*p* ≤ 0.05) between means, as determined by Duncan’s multiple range test

The results of the study on ATP accumulation demonstrated a decrease in ATP over time. When comparing the treatments, it was found that PPCP and MT treatments yielded significantly higher ATP accumulation than the control. Specifically, PPCP treatment demonstrated the highest accumulation of ATP. Similarly, the activity of ATPase decreased over the 28-day storage period. However, the application of 20 μL L^− 1^ PPCP was found to maintain higher activity of ATPase in the tissue of ‘Keitt’ mangoes (Fig. 7a, and 7b).

### Correlation analysis

**Fig. 8:**
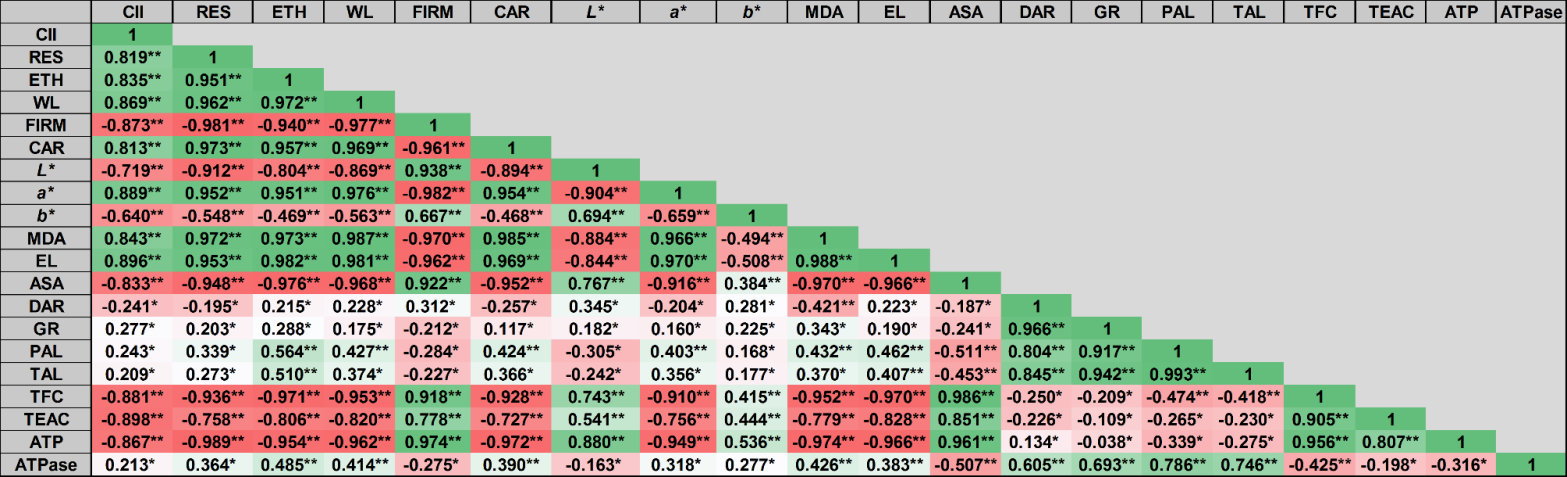
Correlation matrix among several quality attributes of “Keitt” mango fruit in response to PPCP (1-(3-phenyl-propyl) cyclopropane), and melatonin (MT) treatments stored at 5 ± 1 °C for 28 d. Asterisks (^*^ or ^**^) denote statistically significant difference at *P* ≤ 0.05 or 0.01, respectively, in the Pearson correlation analysis, which was performed with three replicates, CII: chilling injury index, RES: respiration rate, ETH: ethylene production, WL: weight loss, FIRM: firmness, CAR: total carotenoid content, MDA: malondialdehyde content, EL: Electrolyte leakage, AsA: ascorbic acid content, DAR: dehydroascorbate reductase, GR: glutathione reductase activity, PAL: phenylalanine ammonia lyase, TAL: tyrosine ammonia lyase, TFC: total flavonoids content, TEAC: trolox equivalent antioxidant capacity

**Fig. 9:**
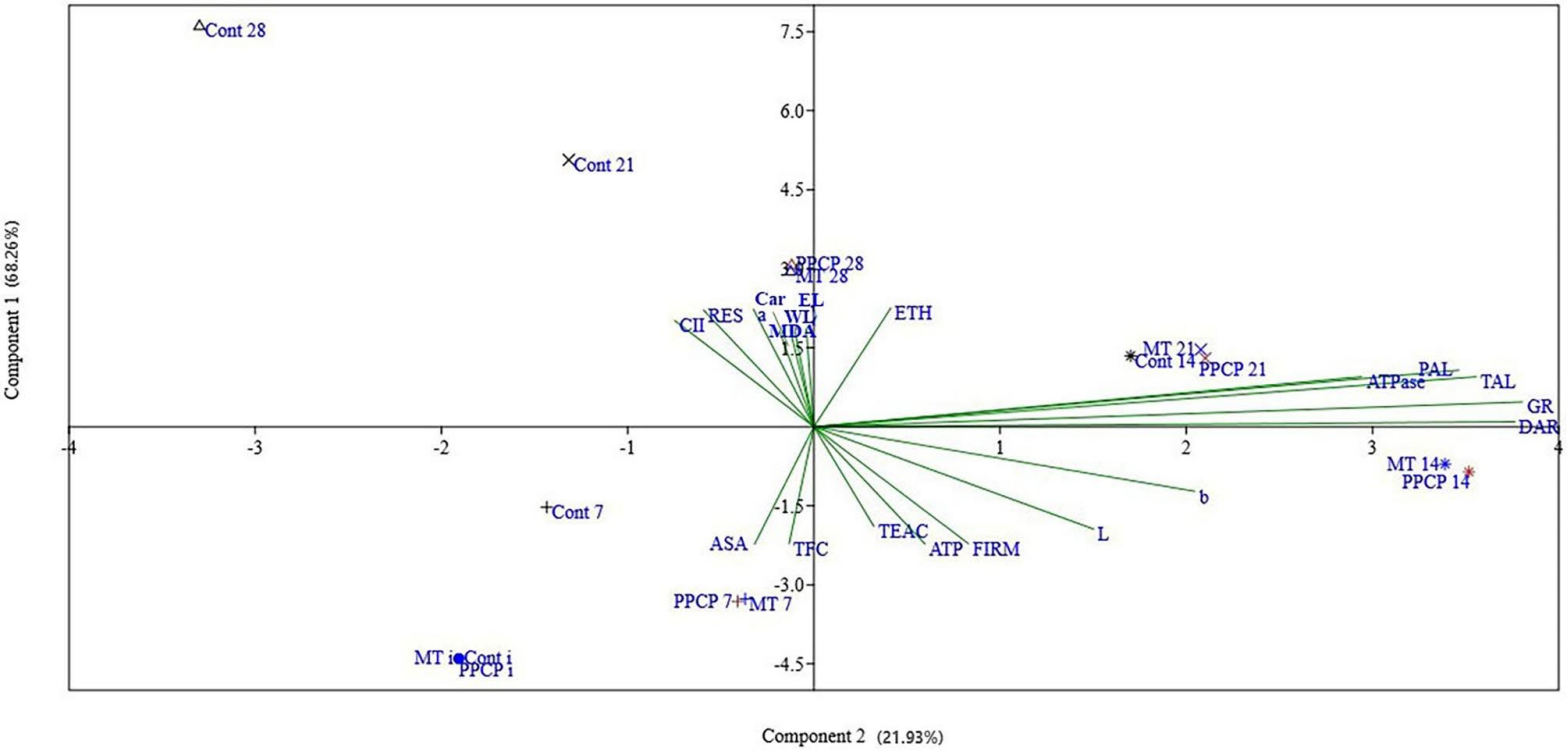
Principal component (PCA) analysis (PC1: 68.26%, PC2: 21.93%) of the quality parameters of “Keitt” mango fruit over 28 d of storage period at 5 ± 1 °C. PPCP (1-(3-phenyl-propyl) cyclopropane), melatonin (MT), Cont: untreated fruit, and numbers (0, 7, 14, 21, and 28) refer to storage days at 5 ± 1 °C, CII: chilling injury index, RES: respiration rate, ETH: ethylene production, WL: weight loss, FIRM: firmness, CAR: total carotenoid content, MDA: malondialdehyde content, EL: Electrolyte leakage, AsA: ascorbic acid content, DAR: dehydroascorbate reductase, GR: glutathione reductase activity, PAL: phenylalanine ammonia lyase, TAL: tyrosine ammonia lyase, TFC: total flavonoids content, TEAC: trolox equivalent antioxidant capacity

The principal component analysis (PCA) and correlation analysis depicted in Figs. 8 and 9 revealed that the first principal component accounted for 68.26% of the variation observed in the “Keitt” mango fruit, while the second principal component accounted for 21.93%. The combined contribution of these two principal components was 90.19%, indicating that these two components could capture most of the differences observed between the control and PPCP and MT treated samples. Furthermore, the multivariate space of PC1 and PC2 illustrated that the storage-induced changes in the fruit quality parameters were influenced differently by the treatments. This was evident in the score plot of the first two principal components, where early storage days (0, 7, and 14) were separated and located on the lower side of the PC1 axis, while the later days (21 and 28) were located on the upper side of the same axis. Additionally, shifts in the PC average values from negative (early days) to positive (later days) were observed in both treated and untreated mango fruit as the storage time progressed.

There is a significant correlation between CI and both respiration and ethylene production clearly indicating an interaction between the role of PPCP and MT as an ethylene antagonist and anti-chilling injury incidence. The untreated fruit (control group) showed a positive correlation with parameters such as the chilling injury index, respiration rate, ethylene production, weight loss, malondialdehyde, and electrolyte leakage. Conversely, these fruits demonstrated a negative correlation with ATP, Trolox equivalent antioxidant capacity, and firmness. As storage time progressed and the values of PC1 shifted from negative to positive, there was a general increase in chilling injury symptoms, as indicated by an increase in the chilling injury index, respiration rate, ethylene production, malondialdehyde, weight loss, and electrolyte leakage, coupled with a decrease in ATP, TEAC, and firmness.

## Discussion

Both PPCP and MT treatments exhibited significant effects on maintaining mango quality and reducing the incidence of CI. The results obtained in this study indicated that the application of PPCP was effective in reducing the respiration rate and ethylene production, while maintaining the ascorbic acid content, total flavonoids, TEAC, DHAR, GR activity, as well as ATP and ATPase levels. Also, the application of MT was effective in reducing weight loss, maintaining firmness, reducing colour changes, decreasing MDA and EL levels, as well as increasing PAL and TAL activity.

The aim of cold storage is to reduce the temperature and slow down the respiration and metabolic processes in fruits, thereby slowing down the ageing process. However, it is important to note that there is a critical threshold beyond which chilling injury may occur. Previous research has reported that storing mango fruit at temperatures below 10–13 °C may result in chilling injury, which can pose a significant problem during marketing and storage, particularly when storing it with other types of fruit [4, 15]. Symptoms of CI in fruits are typically characterized by changes in the colour of the peel, such as darkening or blackening, pitting, and altered ripening patterns. These symptoms may exacerbate when the fruits are removed from low-temperature storage and exposed to room temperature [40].

Fruit chilling injury can have a deleterious influence on fruit metabolism, notably respiration rate and ethylene production [41]. Under chilling stress, respiration, or the process of producing energy, increases; additionally, low temperatures can reduce the production of ethylene, a hormone that plays a key role in fruit ripening and senescence, potentially delaying the ripening process and lowering overall fruit quality [42]. The management of respiration processes and moisture preservation within fruit is critical in determining a range of physiological and biological phenomena that eventually affect the overall quality and shelf-life. Fruit development is marked by a succession of significant changes. Mango respiration rate significantly rises as they ripen [43], and they endure considerable metabolic alterations [44]. In general, it is thought that ethylene aids signal transmission, boosting the expression of genes that encode enzymes responsible for colour changes during ripening and altering flavour, texture, and other properties [45]. Anti-ethylene chemicals serve a variety of purposes. Some of them, such as salicylic acid and 1-methylcyclopropene, block ethylene synthesis and slow the rate of ethylene production. A commonly used endogenous ethylene inhibitor, on the other hand, reduces hormonal action by competing with ethylene for ethylene receptors [46]. Furthermore, ethylene may be converted into CO_2_ utilising exogenous scavenging agents such potassium Permanganate and ozone [47]. According to Sisler et al. [12], cyclopropenes with a methyl group in the 1-position are more efficient than other substituent locations at inactivating the ethylene reaction at lower doses. Among the cyclopropenes with 1-position substituents, 1-ECP and PPCP have been found as especially attractive candidates for practical application [12, 13]. In agreement with previous studies, the use of long chain cyclopropenes as antiethylene, high effectiveness molecules might broaden their utility in agricultural systems [11, 12, 14, 15]. Although both treatments were helpful in lowering the intensity of CI symptoms, the results showed that 200 μM MT for 120 min had a higher impact in reducing CI symptoms. This is consistent with previous studies that found comparable amounts of MT useful in alleviating CI in other fruits such as pomegranate at 100 μM MT [16]. When mango cv. Keitt was moved from cold storage to room temperature, the CI symptoms were much more apparent.

The ethylene production and respiration rate increased progressively under experimental conditions. When compared to the control group, both PPCP and MT therapies were successful in lowering this rise. Although these rates were lower than those observed in previous studies such as Khedr [30] at higher storage temperatures, the 20 μL L^− 1^ PPCP treatment was found to be the most effective in reducing both the respiration rate and ethylene production at 28 d of observation when compared to other treatments. It is worth mentioning that cyclopropene compounds have been known for their potency as ethylene antagonists [11, 12, 48]. The use of PPCP at a concentration of 20 μL L^− 1^ was found to dramatically slow the rate of fruit ethylene generation and respiration. Despite being evaporated to produce a gaseous form of PPCP, the combination was still efficient in blocking ethylene activity on fruit. When applied to fruit, PPCP contains an aryl ring, has been demonstrated to be effective in preventing ethylene activity [49]. Both PPCP and MT were shown to reduce the rate of respiration and ethylene generation in fruit, although PPCP had a stronger impact. This may be due to its direct influence on ethylene receptor binding and inhibiting ethylene binding and subsequent activity [15, 42].

Firmness is an important quality attribute for many fruits since it affects customer acceptability as well as the fruit’s capacity to endure handling and transportation. Fruit chilling damage can have a negative impact on fruit firmness. Physical changes in fresh mango fruit, such as softening, are critical for handling, storage, and customer approval [50]. Low temperatures associated with CI can damage the cell membrane and other biological components, leading to water and other substances being lost from the fruit [31]. This can result in a reduction in turgor pressure, which is responsible for the fruit firmness. The fruit may have a softer feel when turgor pressure diminishes. Furthermore, low temperatures can influence the activity of enzymes and other metabolic processes within the fruit, causing the disintegration of cell walls and other structural components and contributing to a loss of firmness. Fruit firmness did progressively diminish under all conditions, but fruit treated with 200 μM MT and 20 μL L^− 1^ PPCP were much firmer than control fruit, which showed greater softening rates (Fig. 3a). In terms of storage durations, both PPCP and MT revealed convergent results. Several investigations have shown that MT and cyclopropane molecules have a function in controlling respiration rates and reducing hydrolytic enzymatic activity in cell walls [32, 51]. MT has been demonstrated to efficiently suppress the activities of pectin methylesterase and polygalacturonase, both of which are known to play important roles in fruit softening [52].

Low temperatures destroy the cell membrane and other cellular components, causing water and other substances to flow from the fruit. This, in turn, causes a reduction of turgor pressure, which is responsible for keeping the fruit firm, as well as a drop in fruit weight. Fruit weight loss is an important measure of both fruit quality and freshness retention. Fruit weight loss is mostly driven by water loss during the respiration and transpiration processes [53]. The negative impacts of weight loss are not just confined to economic loss, but also to a fall in water content, which results in unfavorable alterations such as shrinking of the fruit exocarp and a decrease in nutritional value. Furthermore, water loss is strongly connected to several metabolic activities that occur after harvest and throughout storage. Several studies have shown that respiration rate is important in weight reduction [31]. Furthermore, it can have an impact on the nutritional value of the fruit because weight reduction is frequently followed by a fall in sugar content and other nutrients. Furthermore, all treatments utilized reduced weight loss (percentage) compared to the control, with the control group fruit losing twice as much as the melatonin-treated fruit after 28 days of storage under experimental conditions (Fig. 2d). The results are comparable with those reported by Liu et al. [1] using 0.5 mM MT on ‘Guifei’ mangoes for 1 h.

Fruit chilling injury can also influence the colour of the mango skin. One of the symptoms of CI is the emergence of a light or yellow discoloration on the peel caused by pigment breakdown. Epicarp browning is associated with decreases in L^*^ values and changes in chromaticity characteristics [54]. When mango cells are subjected to low temperatures, the chlorophyll can degrade, leading in the loss of green colour and the appearance of a pale or yellow discoloration. Furthermore, low temperatures can influence other pigments found in mango peel, such as carotenoids, which are responsible for the fruit’s yellow colour [1]. The fundamental determinant of the different flesh colour of mango fruit is carotenoid pigments. When determining the ripeness or quality of mango fruit, it is critical to examine colour by extracting carotenoid pigments or using a colourmeter. In general, mango fruit flesh colour is a more trustworthy predictor than peel colour [44]. As the fruit grew, the golden colour increased consistently. All treatments showed skin colour alterations that occurred later than the control, which was consistent with Kittur et al. [55] findings. Furthermore, Liu et al. [1] discovered a link between fruit ripening and variations in mesocarp colour. Low temperatures can impede carotenoids production, resulting in a reduction in total carotenoid content. Furthermore, low temperatures can induce cell and membrane damage, resulting in the leaking of carotenoids and other chemicals from the fruit, exacerbating the drop in total carotenoid concentration. The total carotenoid concentration is an important quality factor for many fruits since it is directly connected to nutritional value. The use of PPCP and MT has been found to successfully control colour changes and postpone the ripening process. This treatment method not only provides cosmetic benefits, but it also protects against symptoms related to chilling disorder.

MDA is a byproduct of lipid peroxidation, which is a process in which reactive oxygen species (ROS) assault the cell membrane and destroy the lipids. MDA is a frequently used measure of cellular lipid peroxidation and oxidative stress. The higher the MDA concentration, the more severe the oxidative stress and cell membrane damage. Electrolyte leakage is a measure of the cell membrane’s integrity. Electrolyte leaking from fruit cells is utilized as a diagnostic method to determine cell membrane damage [56]. When compared to control samples, fruit treated with PPCP and MT and kept at suboptimal temperatures exhibits less electrolyte loss and hence less chilling damage [56]. Furthermore, preconditioning zucchini fruit by slow temperature reduction has been observed to result in lower ion leakage and a commensurate improvement in membrane integrity, resulting in a significant reduction in chilling damage [57]. When the cell membrane is broken, the electrolytes contained inside the cell (such as potassium, sodium, and chloride) might leak out, increasing the concentration of these ions in the surrounding medium [58–60]. PCA analysis demonstrated a high association between MDA, EL, peel and pulp colour, and the frequency of chilling damage in mango fruit. As a result, these measures may serve as critical markers for the occurrence of chilling damage in mangoes.

Ascorbic acid levels and related enzymes may be affected by chilling disorder. Ascorbic acid is an important antioxidant that improves the overall quality of the fruit. The low temperatures associated with CI can destroy cells and membranes, resulting in ascorbic acid and other chemicals leaking from the fruit. As a result, the overall concentration of ascorbic acid may decrease. In addition to lowering ascorbic acid concentration, CI can affect the activity of enzymes involved in ascorbic acid metabolism. Specific enzymes, such as ascorbate oxidase, L-galactono-1,4-lactone dehydrogenase, and D-galacturonate dehydrogenase, aid in the breakdown of ascorbic acid in fruit. These enzymes are in charge of degrading ascorbic acid and lowering their concentration over time [61]. Ascorbic acid regulates the concentration of ROS via participating in the AsA-GSH cycle, which works to counteract oxidative stress. Key enzymes such as APX, DHAR, GR, and monodehydroascorbate reductase all have an impact on the AsA-GSH cycle [62]. Chilling injury can also impact the fruit flavonoid concentration. Flavonoids are polyphenolic compounds present in a variety of fruits and vegetables, including mango. When mango cells are subjected to low temperatures, the production of flavonoids is reduced, resulting in a drop in flavonoid concentration. Furthermore, low temperatures can damage cells and membranes, resulting in flavonoids and other chemicals leaking from the fruit. This may contribute to a drop in flavonoid content.

Fruit should be maintained at temperatures above the chilling temperature range to limit the loss of flavonoids and other CI symptoms, and suitable packing and handling should be utilised to prevent damage to the fruit during shipping and storage [63]. The dissociation of the enzymatic defence system (DHAR, GR, and ATPase) in tissue and with CI suggested that this was the primary mechanism of CI reduction in mangoes regardless of treatment. Although studies have linked DHAR, GR, and ATPase activities to decreased oxidative damage produced by CI in mangoes [64], the non-enzymatic defence mechanism (AsA and TFC) should also be evaluated, since the mango showed no oxidative damage over the storage period [59, 60, 65]. TEAC is an antioxidant capacity measure that quantifies a substance’s ability to neutralise free radicals, which are chemicals that can cause cell damage and contribute to the development of many disorders. The antioxidant activity of trolox, a water-soluble homologue of vitamin E, is used to calculate TEAC [66]. DHAR and GR are enzymes that play key functions in the antioxidant defence system of cells [40, 67]. DHAR catalyses the conversion of dehydroascorbate to ascorbate, a key antioxidant in the cell. While GR catalyses the conversion of oxidised glutathione (GSSG) to reduced glutathione (GSH), both act as antioxidants in the cell. ATP (adenosine triphosphate) is usually referred to be the “energy currency” of cells. It stores energy in high-energy bonds, which may be broken down when needed to release energy. ATPase is an enzyme that catalyses the hydrolysis of ATP, which is the process of releasing energy by breaking the links between the phosphate groups in the molecule. There are several varieties of ATPase enzymes, including Na^+^/K^+^ ATPase, H^+^ ATPase, and Ca^2+^ ATPase, each with a distinct purpose in the cell [67].

PAL and TAL are enzymes that catalyze the initial step in the production of phenylpropanoids, a class of secondary metabolites that also includes flavonoids and tannins [68, 69]. PAL catalyzes the conversion of phenylalanine to trans-cinnamic acid, a flavonoid biosynthesis step. TAL catalyzes the conversion of tyrosine to p-coumaric acid, a step in flavonoid biosynthesis. Both PAL and TAL activity are thought to be indicators of the plant’s defensive system. They oversee the synthesis of diverse phenolic compounds, which function as antioxidants and antibacterial agents, which correspond to the enzymatic activity of other enzymes within the fruit, such as polyphenol oxidase [70]. PAL and TAL activity can be measured using spectrophotometry or fluorometry methods. Environmental variables like abiotic stress, disease infection, and low temperature exposure can all influence the activity of these enzymes. High PAL and TAL activity is related to a robust defense system and a decreased vulnerability to stress and disorder, whereas low activity may suggest a poor defense system and a higher susceptibility to stress and disease.

## Conclusions

The utilization of treatments with 20 μL L^− 1^ of PPCP or 200 μM MT was found to effectively preserve the postharvest quality parameters, in terms of bioactive compounds, energy metabolism, and antioxidant activity, of mangoes cv. “Keitt” that were stored at 5 ± 1 °C for 28 d. Based on the results, it seems that ethylene production is involved in chilling tolerance in mango fruit, because as an ethylene antagonist, PPCP can reduce CI in mangoes. Melatonin is also observed to inhibit ethylene production in this study, which can be inferred that the decreased chilling incidence in MT-treated fruit is also associated with the inhibited ethylene production. This was attributed to the preservation of membrane integrity, which resulted from low production of MDA and electrolyte leakage. These treatments also delayed senescence by inhibiting ethylene production and the respiration rate, which are linked to the natural ripening and senescence process. The low level of oxidative stress observed was due to the stimulation of enzymatic and non-enzymatic antioxidant systems in the mango fruit. Therefore, the PPCP and MT treatments were able to alleviate the symptoms of chilling injury in mangoes. These findings demonstrate the potential of PPCP and MT as effective treatments to preserve mango quality and reduce post-harvest losses. Further investigations are recommended to determine the optimal concentrations and application methods for these treatments, as well as to explore their potential effects on other fruit and vegetable crops.

## Data Availability

The original contributions presented in the study are included in the article, and further inquiries can be directed to the corresponding author.
